# Comment On: Valette et al. Melanocortin-4 Receptor Mutations and Polymorphisms Do Not Affect Weight Loss after Bariatric Surgery. *PLOS ONE* 2012; 7(11):E48221

**DOI:** 10.1371/journal.pone.0093324

**Published:** 2014-03-31

**Authors:** David Meyre, Philippe Froguel, Fritz F. Horber, John G. Kral

**Affiliations:** 1 Department of Clinical Epidemiology and Biostatistics, McMaster University, Hamilton, Ontario, Canada; 2 Centre National de la Recherche Scientifique (CNRS) Unité Mixte de Recherche (UMR) 8199, Lille Pasteur Institute, Lille, Nord, France; 3 Department of Genomics of Common Disease, Imperial College London, London, United Kingdom; 4 Department of Internal Medicine, Landesspital, Vaduz, Liechtenstein; 5 University of Berne, Berne, Switzerland; 6 Department of Surgery, State University of New York Downstate Medical Center, Brooklyn, New York, United States of America; University of Texas Health Science Center at San Antonio, United States of America

## To the Editor

We read with interest the *PLOS ONE* article by Valette et al. in 2012 assessing associations between gene variations at the melanocortin 4 receptor (MC4R) locus and weight loss after bariatric surgery in a French longitudinal cohort [1]. We were the first to describe effects of *MC4R* gene variations on outcomes of bariatric surgery [2] and have several concerns about the current study.

There seems to be a genotyping error of the rs17782313 variant downstream of *MC4R*. As the authors acknowledge, the minor allele frequency (MAF) of this variant was 0.27 in 1443 Swedish bariatric surgery patients [3] which is comparable to the MAF of 0.30 we found in 1274 severely obese similar Swiss patients [4]. In the presence of Hardy-Weinberg equilibrium, MAF of 0.27 for the variant rs17782313 should yield 345 homozygous TT carriers, 256 heterozygous TC carriers and 47 homozygous CC carriers among the 648 patients described by Valette et al. [1]. However, their genotypic distribution is 641 TT, 4 TC and 3 CC carriers implying an error in their genotype count. There are various causes of genotyping artifacts including poor DNA quality, genotyping of different polymorphisms, technical faults, subjective genotype classification, and human errors during genotyping or data transfer [5]. In the absence of information about the precise method used to genotype the rs17782313 polymorphism in the paper it is difficult to assess potential causes of the discordant genotype distribution. The authors failed to describe any quality control procedures to ensure the integrity of their genotyping such as: call rate, Hardy-Weinberg equilibrium test, double-genotyping concordance rate and comparison of the MAF with published databases in comparable ethnic groups [5].The very significant departure of the genotypic distribution of the variant rs17782313 described by Valette et al from Hardy-Weinberg equilibrium (P<10^−20^) suggests an error in technique.

We fully acknowledge the value of a matched case control design to longitudinally study responses to bariatric surgery, but such design requires sufficient phenotypic data to interpret outcomes. Valette *et al.* did not seem to assess associations between *MC4R* genetic variants with preoperative metabolic parameters known to affect surgical outcomes. Potentially, relevant metabolic phenotypic differences at baseline between carriers and non-carriers of *MC4R* genetic variants might have clarified the findings of their study leading to different conclusions.

The authors mentioned that the analysis of a ‘large group of *MC4R* mutation carriers’ was one of the strengths of their study. We respectfully disagree with this statement. On the contrary, the small number of subjects in the different *MC4R* genotype groups (e.g. 9 carriers of loss-of-function mutations or 10 carriers of gain-of-function mutations) constitutes a major limitation of this study. The fact that the authors only demonstrated a nominally significant association between the more prevalent *MC4R* genotype group (22 carriers of the −178 A/C polymorphism) and surgical outcomes argues for an overall lack of statistical power in the study.

Lastly, the stated purpose of the paper was to evaluate surgical weight loss in patients with MC4R mutations and polymorphisms. Unfortunately the authors chose to do so as early as 12 months postoperatively, before the majority of patients reach weight loss nadir. The rapid weight loss phase after gastric banding and bypass is approximately 9–18 months, with differences in trajectory between the two [6]. By studying patients before they reached a steady, homeostatic state, Valette et al. abrogated their ability to analyze relationships between genetic variants and complications, reoperations, clinically meaningful effects on serious comorbidities or weight regain, altogether undermining the relevance of the study.
